# Continuous Decoding of Hand Movement From EEG Signals Using Phase-Based Connectivity Features

**DOI:** 10.3389/fnhum.2022.901285

**Published:** 2022-06-30

**Authors:** Seyyed Moosa Hosseini, Vahid Shalchyan

**Affiliations:** Neuroscience and Neuroengineering Research Lab, Department of Biomedical Engineering, School of Electrical Engineering, Iran University of Science and Technology, Tehran, Iran

**Keywords:** brain computer interface (BCI), phase-locking value (PLV), magnitude-squared coherence (MSC), electroencephalography (EEG), multiple linear regression (MLR), trajectory decoding

## Abstract

The principal goal of the brain-computer interface (BCI) is to translate brain signals into meaningful commands to control external devices or neuroprostheses to restore lost functions of patients with severe motor disabilities. The invasive recording of brain signals involves numerous health issues. Therefore, BCIs based on non-invasive recording modalities such as electroencephalography (EEG) are safer and more comfortable for the patients. The BCI requires reconstructing continuous movement parameters such as position or velocity for practical application of neuroprostheses. The BCI studies in continuous decoding have extensively relied on extracting features from the amplitude of brain signals, whereas the brain connectivity features have rarely been explored. This study aims to investigate the feasibility of using phase-based connectivity features in decoding continuous hand movements from EEG signals. To this end, the EEG data were collected from seven healthy subjects performing a 2D center-out hand movement task in four orthogonal directions. The phase-locking value (PLV) and magnitude-squared coherence (MSC) are exploited as connectivity features along with multiple linear regression (MLR) for decoding hand positions. A brute-force search approach is employed to find the best channel pairs for extracting features related to hand movements. The results reveal that the regression models based on PLV and MSC features achieve the average Pearson correlations of 0.43 ± 0.03 and 0.42 ± 0.06, respectively, between predicted and actual trajectories over all subjects. The delta and alpha band features have the most contribution in regression analysis. The results also demonstrate that both PLV and MSC decoding models lead to superior results on our data compared to two recently proposed feature extraction methods solely based on the amplitude or phase of recording signals (*p* < 0.05). This study verifies the ability of PLV and MSC features in the continuous decoding of hand movements with linear regression. Thus, our findings suggest that extracting features based on brain connectivity can improve the accuracy of trajectory decoder BCIs.

## Introduction

Brain computer interfaces (BCIs) are systems based on signal processing techniques that translate the brain's electrical activity into control commands (Wolpaw and Wolpaw, 2012; Rao, 2013). The earlier generation of BCIs aimed to classify different brain states to generate discrete commands (Wolpaw et al., 1991; McMullen et al., 2013). Although the classification-based BCIs work appropriately for discrete tasks such as spellers or virtual keyboards (Rezeika et al., 2018), they are not suitable for tasks involving continuous parameters estimation such as human hand movement. Many studies have focused on decoding continuous movement from brain activities for this sake. Researchers in the realm of invasive BCIs were the first to decode hand movement parameters continuously from primates and humans using spiking activity (Taylor et al., 2002; Kim et al., 2008; Hochberg et al., 2012), local field potential (LFP) (Mehring et al., 2003; Flint et al., 2012), and electrocorticography (ECoG) signals (Shimoda et al., 2012; Nakanishi et al., 2013). This new approach is consistent with the continuous nature of human motion. However, the invasive recording of brain activity involves great concerns such as scarring brain tissues, post-operation infections and signal reliability over time. Hence, the usage of invasive BCIs is still limited. Therefore, there is a need for BCIs based on non-invasive recording modalities such as EEG to decode continuous movements.

Employing non-invasive recording modalities was ignored until recently when Bradberry et al. (2010) reported the feasibility of decoding hand velocity in a 3D center-out reaching task using low-delta EEG signals. Kim et al. (2014) also exploited low-frequency EEG signal amplitudes to decode two types of hand trajectories in execution and observation/imagination tasks. Korik et al. (2016, 2018) challenged the common belief that only the low-delta band of EEG carries information about continuous decoding of hand kinematics and claimed that mu (8–12 Hz) and beta (12–28 Hz) bandpowers encode information for hand motion trajectory prediction. Additionally, the role of delta and beta oscillatory rhythms in decoding hand velocity in a drawing task was demonstrated by Lv et al. (2010).

The most common features that have been used for decoding hand trajectory are amplitude-related features such as power in various frequency bands. In contrast, the possibility of using brain connectivity features is still almost unexplored. There are some limited studies in this area as far as we know. Benz et al. (2012) exploited time-varying dynamic Bayesian networks (TV-DBN) to extract features from ECOG signals for predicting joint angle in a palmar grasp task. Li et al. (2018) have proposed using connectivity analysis from brain functional network (BFN) alongside a hierarchical linear model (HLM) to decode continuous positions of spiral hand movement from EEG recordings. The effectiveness of phase-locking value (PLV) features of EEG signals in the binary classification of executed or imagined hand movement was investigated in Chouhan et al. (2018) and Benzy and Vinod (2019), respectively. However, as far as we know, the literature has not examined the viability of using EEG phase-based connectivity features in the continuous decoding of hand kinematics during executed movement tasks.

To address this issue, we designed a cue-based 2D center-out task in which a healthy subject performed hand-reaching movement in a horizontal plane toward four targets. In one trial of this task, the hand movement cycle comprised three phases: moving from the origin to one of four targets, pausing for 400 ms at the target, and returning to the first place. The EEG signals and positions of the right hand were recorded simultaneously during the task. Phase locking value (PLV) and magnitude-squared coherence (MSC) were extracted from EEG signals as predictors for multiple linear regression (MLR) to decode hand positions. To the best of our knowledge, this work is the first to investigate the feasibility of using phase-based connectivity in decoding continuous hand trajectory from EEG signals.

The rest of the paper is organized as follows: Section Materials and Methods is dedicated to the methods and materials. The results are presented in Section Results. Finally, the discussion is left for Section Discussion.

## Materials and Methods

This study investigates the feasibility of decoding continuous hand trajectory using PLV and MSC features in a 2D center-out task from EEG signals. Recording signals and designing the experiment will be discussed in the following subsections.

### Subjects and Equipment

Seven healthy male subjects aged between 25 and 35 participated in the study. All subjects were right-handed. The experiment was done in one session for each subject. The study meets the recommendations of the Ethics Committee of the Iran University of Science and technology. All subjects, before the participation, gave written informed consent following the Declaration of Helsinki. The protocol was approved by the Ethics Committee of Iran University of Medical Sciences with approval ID IR.IUMS.REC.1400.382.

**Figure 1:**
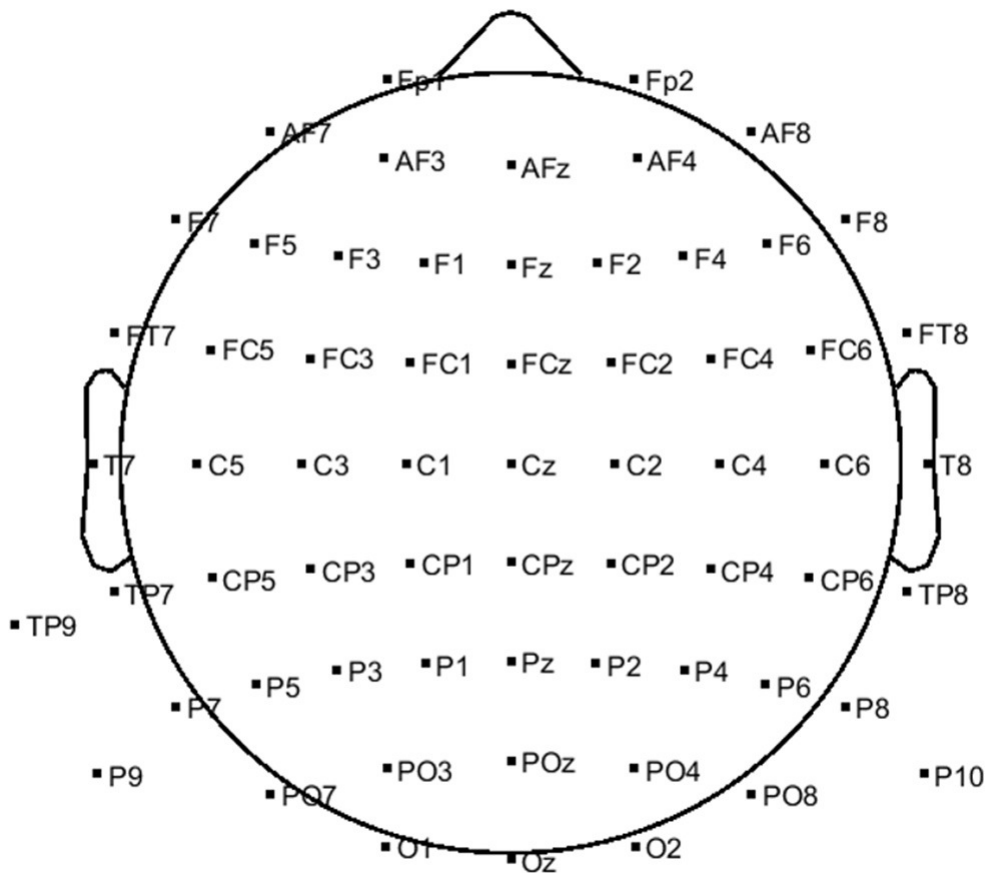
Positions of 63 active EEG electrodes on the scalp.

The EEG signals were recorded *via* a g.HIamp system (g.tec, GmbH, Austria) with 63 active electrodes at a 512 Hz sampling rate. The electrodes were placed over the scalp with the distribution depicted in Figure 1. Reference and ground electrodes were placed at the right mastoid and Fpz locations, respectively. Three additional passive electrodes were placed at the superior, inferior, and outer canthi of the right eye to record EOG activities. The right-hand position was simultaneously recorded *via* the Leap Motion device (Leap Motion, Inc.). The sampling rate of Leap Motion was 115 Hz. To eliminate the motion sensor noise, the output signal of leap motion was filtered with a fourth-order zero-phase Butterworth low-pass filter at 1.5 Hz.

### Experimental Task

**Figure 2:**
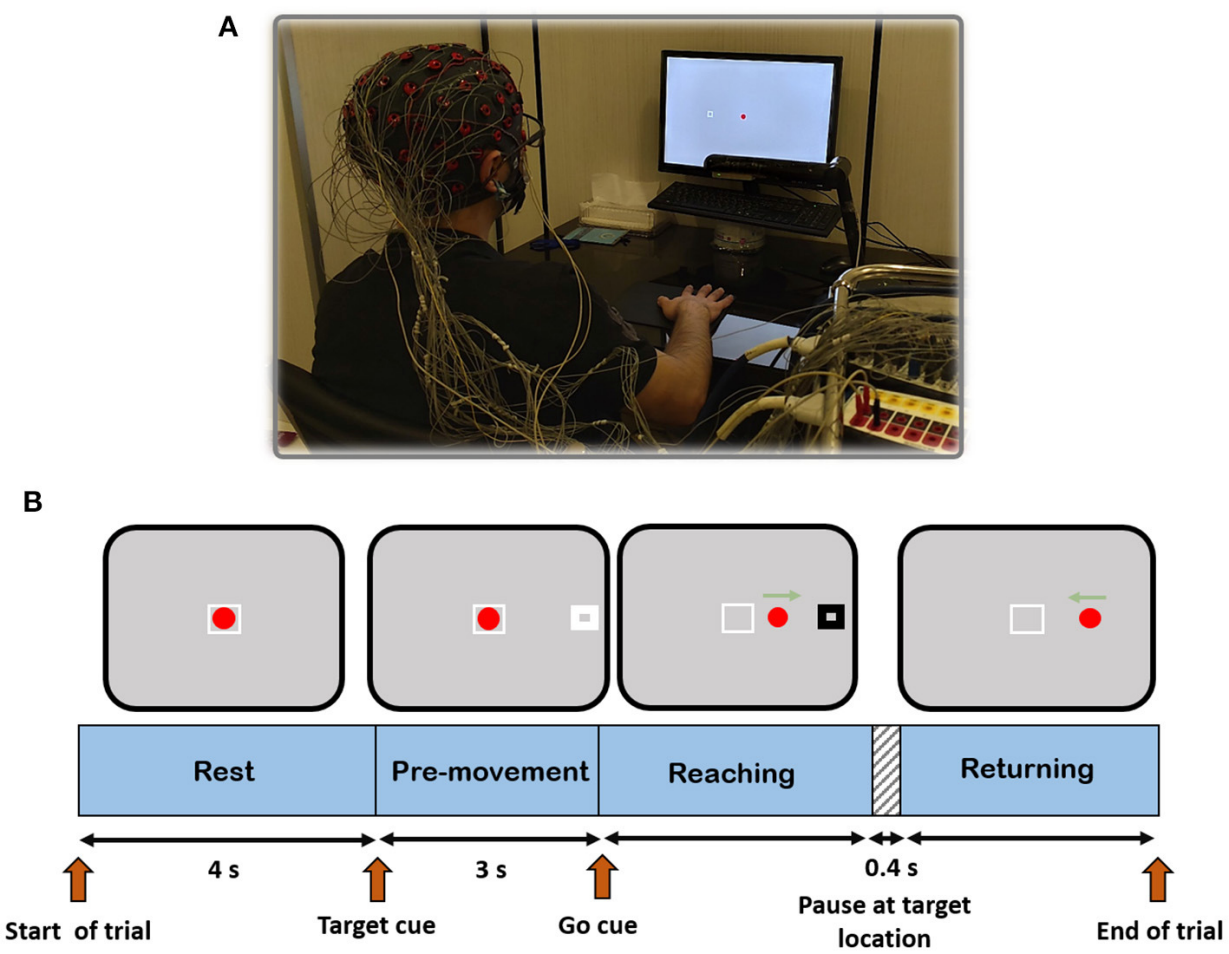
**(A)** Experimental setup showing a subject performing the task. **(B)** Task protocol timeline.

In order to analyze the main objective of this work, a cue-based 2D center-out right-hand movement task with four targets was designed. Reaching each target required rightward, leftward, upward, and downward hand movement from a center position called home to one of the four orthogonal directions in the horizontal plane. The experimental setup and the timeline for each trial are depicted in Figure 2. The experiment could be described as follows: the subject sat in front of a computer screen at a 50 cm distance. The leap motion device was mounted on a leg ~25 centimeters above the table surface. The subject's palm position of the right hand was mapped into a red circle at the home position on the screen. Each trial began with a 4 s rest. After that, a target (white rectangle) appeared at one of four target positions 10 cm away from the home position. After 3 s, the white rectangle turned black (go-cue), and the subject started moving his hand toward the target. When the red circle touched the target, the target rectangle turned white, and after 400 ms, it disappeared (go back cue). Then, the subject returned the red circle to the home position to end the trial. Each trial includes three parts: reaching toward the target, pausing at the target location, and returning. The rationale behind the 400 ms pause at the target place was to prevent the subject from rushing into the task and making the movement smooth. If the subject could not complete a trial in 10 s, the trial was discarded.

The experiment was conducted in one session for seven male subjects. Each session was composed of four blocks consisting of 40 trials. The subject had to move toward each target ten times in each block. The order of presenting targets to subjects was random in each block. A break interval of 2–3 min was applied between the recording blocks. Overall, each subject performed 160 trials. The average trial duration was 4.16 ± 0.64 (mean ± SD) s.

### Data Preprocessing and Feature Extraction

All EEG signals were highpass filtered at 0.2 Hz with a fourth-order zero-phase Butterworth filter. The power line frequency at 50 Hz was notch-filtered. All EOG channels were passed through a bandpass filter (0.5–5 Hz, fourth-order zero-phase Butterworth) (Klados et al., 2011). The independent component analysis (ICA) was employed to remove the eye, heart, and muscle-related artifacts (Delorme and Makeig, 2004). The ICA components with a correlation of more than 0.4 with recorded EOG signals were considered as eye artifacts. The artifact-related components were removed, and then the EEG signals were reconstructed from the remaining components. In addition, we eliminated the seven most frontal electrodes in the Fp and AF rows. Hence, 56 electrodes remained for feature extraction and movement decoding analysis. In each trial, the hand position signals were upsampled to 512 Hz to have the same length as corresponding EEG signals. All data processing was carried out with MATLAB 2019b (The MathWorks, Inc.).

One of the great challenges of calculating brain connectivity from EEG signals is the effect of volume conduction which can produce spurious connectivity between electrodes (Cohen, 2014). To address this issue, the surface Laplacian filter (Perrin et al., 1989) was applied to the EEG signals to attenuate the effect of volume conduction and therefore prepare the data appropriately for connectivity analyses. It also improved the spatial resolution of EEG signals over the scalp (Carvalhaes and De Barros, 2015).

**Figure 3:**
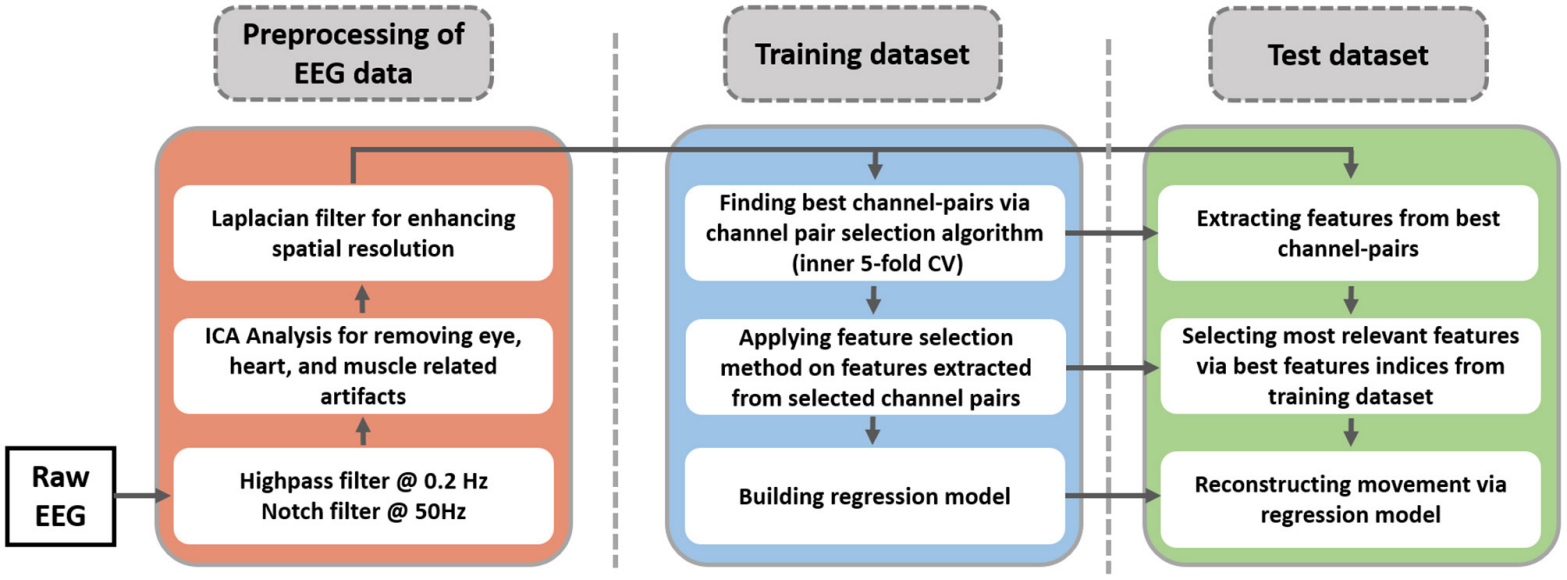
Schematic of proposed EEG data processing pipeline. The blue and the green parts indicate the training and test dataset of outer cross-validation (10-fold CV).

In this study, an inner-outer (nested) cross-validation (CV) scheme has been exploited for channel-pair selection, feature selection, and model evaluation (Chouhan et al., 2018; Korik et al., 2018). The inner-outer CV allows selecting and optimizing a set of parameters using the inner fold CV and calculating the final prediction in outer folds based on optimal parameters that are selected by the inner CV. In this work, ten outer folds and five inner folds were used. In other words, the result of regression analysis is calculated based on ten folds CV. The training dataset of this CV is split into five folds for selecting the best channel pairs and feature selection. After finding the best channel pairs, a feature selection algorithm is applied to the features extracted from selected channel pairs. The indices of best channel pairs and movement-related features in the training dataset will be used on the test dataset. The proposed methodology for hand movement reconstruction is illustrated in Figure 3. The results of the study are reported for ten repetitions of the nested CV.

The objective of this study was to investigate the feasibility of using two types of phase-based connectivity, PLV, and MSC, to decode hand positions in a center-out task. First, the definition of PLV and MSC is presented. The PLV between two signals is a measure of phase synchrony between them (Bastos and Schoffelen, 2016). In fact, PLV calculates the mean of the phase difference between two signals over time or trials. The two signals must be represented in the analytic form (phase and amplitude), which is usually calculated *via* the Hilbert transform. The mathematical formulation of PLV is defined as:

(1) $$\begin{array}{r} PL{V\left(f\right)}_{xy}=|n^{-1}\overset{n}{\underset{t=1}{\sum }}e^{j\phi_{xy}\left(t,f\right)}| \end{array}$$

where *x*(t), *y*(*t*) represent two analytic signals and ϕ_*xy*_(*t, f*) indicates the phase difference between them at frequency *f*. The average phase difference is calculated over *n* samples.

The MSC can be viewed as a type of PLV that considers the amplitude of analytic signals. The mathematical representation of MSC is described as follows:

(2) $$\begin{array}{r} MSC{\left(f\right)}_{xy}=|\frac{{S\left(f\right)}_{xy}}{{S\left(f\right)}_{xx}{S\left(f\right)}_{yy}}| \end{array}$$

where *S*(*f*)_*xy*_ represents cross-spectral density between signals *x*(t) and *y*(*t*). Autospectral densities of signal *x*(t) and *y*(*t*) at frequency *f* are denoted by *S*(*f*)_*xx*_ and *S*(*f*)_*yy*_. The cross-spectral and autospectral densities are formulated as follows:

(3) $$\begin{array}{r} {S\left(f\right)}_{xy}=|n^{-1}\overset{n}{\underset{t=1}{\sum }}|m_{x}\left(t,f\right)||m_{y}\left(t,f\right)|e^{j\phi_{xy}\left(t,f\right)}{|}^{2} \end{array}$$

(4) $$\begin{array}{r} {S\left(f\right)}_{xx}=n^{-1}\overset{n}{\underset{t=1}{\sum }}|m_{x}\left(t,f\right){|}^{2} \end{array}$$

(5) $$\begin{array}{r} {S\left(f\right)}_{yy}=n^{-1}\overset{n}{\underset{t=1}{\sum }}|m_{y}\left(t,f\right){|}^{2} \end{array}$$

where *m*_*x*_(*t, f*) and *m*_*y*_(*t, f*) are the amplitude of analytic signals *x*(*t*) and *y*(*t*) at frequency *f*. Analytic representation of a narrowband signal such as *u*(*t*) can be obtained *via* Hilbert transform:

(6) $$\begin{array}{r} z\left(t\right)=u\left(t\right)+j\text{HT}\left(u\left(t\right)\right) \end{array}$$

where **HT**(*u*(*t*)) denotes the Hilbert transform of signal *u*(*t*) defined as:

(7) $$\begin{array}{r} \text{HT}\left(u\left(t\right)\right)=u\left(t\right)*\frac{1}{\pi t} \end{array}$$

where * represents the convolution operation between two time series. The instantaneous phase

sequence ϕ_*u*_(*t*) is defined as the angle of the analytic signal:

(8) $$\begin{array}{r} \phi_{u}\left(t\right)=\;\arctan\left(\frac{\text{HT}\left(u\left(t\right)\right)}{u\left(t\right)}\right) \end{array}$$

The Hilbert transform requires the signal to be narrowband to make correct phase predictions. In other words, the phase of the signal with broadband frequency content cannot be estimated accurately (Boashash, 1992). Hence, in order to calculate PLV and MSC, 30 second-order Butterworth filters with 1.5 Hz bandwidths were designed. The center frequency of each narrowband filter was uniformly chosen from the interval 1-45 Hz. the spectral content of each EEG channel was split into 30 sub-bands using this filter bank.

In order to extract features, the channel pairs with the highest correlation with movement along the x-axis and y-axis were selected. The algorithm for channels pairs selection will be discussed in Section Channel Pair Selection. The PLV and MSC measures were calculated in 30 sub-bands using a one-second sliding window (512 samples) between each selected channel pair. The time window moved along signals with a 125 ms stride (64 samples). Therefore, after feature extraction, the sampling rate was equal to $\frac{512}{64}=8\;$Hz. This procedure was applied to 40 pairs of channels representing the highest correlation with hand motions. The corresponding hand motion position signals were downsampled accordingly to reach a sampling rate of 8 Hz. We also considered six time-lags of signals (0, 125, 250, 375, 500, and 625 ms). So, the feature space contained 30 × 40 × 6 = 7,200 features. This feature space was colossal and led to the overfitting problem. Hence, a feature selection algorithm was exploited to select the best feature subset and alleviate the effect of the overfitting problem. The feature selection method will be explained in Section Feature Selection.

### Decoding Model

The multiple linear regression (MLR) model has been exploited to decode the right hand's position in two dimensions. The mathematics of MLR is straightforward. It attempts to linearly combine features (regression predictors) to reconstruct the response variable (hand position). MLR model is defined as follows:

(9) $$\begin{array}{r} y_{MLR}\left(t\right)=\beta_{0}+\overset{N_{c}}{\underset{c=1}{\sum }}\overset{N_{f}}{\underset{i=1}{\sum }}\overset{N_{\tau }-1}{\underset{\tau =0}{\sum }}\beta_{ci\tau }x_{ci}\left(t-\tau \right)+\;\epsilon \left(t\right) \end{array}$$

where *y*_*MLR*_(*t*) is the estimation of actual hand position *y*(*t*), β_*ciτ*_ indicates the weights of regression and ϵ(*t*) is the Gaussian noise with zero mean and σ^2^ variance representing the error of the model. The intercept is indicated by β_0_. The *N*_*c*_, *N*_τ_, and *N*_*f*_ denote the number of channel pairs, signal lags, and frequency sub-bands, respectively. The above equation can be rewritten in the matrix format:

(10) $$\begin{array}{r} Y_{MLR}=X.\beta +\;\epsilon \end{array}$$

where matrix $X\in R^{n\times \left(p+1\right)}\;$denotes the predictor matrix with *n* time samples and *p* = *Nc* × *N*_*f*_ × *N*_τ_ predictor variables. The columns of *X* contain the time-resolved PLV or MSC features with their lags. An extra column of ones is included to introduce the intercept term. By employing the least-squares method, the solution for β is straightforward:

(11) $$\begin{array}{r} \beta ={\left(X^{T}X\right)}^{-1}X^{T}Y \end{array}$$

In this study, the metric exploited to assess the model accuracy is the Pearson correlation. It is very common to employ this metric in hand trajectory decoding studies (Bradberry et al., 2010; Úbeda et al., 2017; Mondini et al., 2020). For two random variables *X*, and *Y*, the Pearson correlation coefficient (PCC) is defined as:

(12) $$\begin{array}{r} PCC\left(X,Y\right)=\frac{cov\left(X,Y\right)}{\sqrt{var\left(X\right).var\left(Y\right)}} \end{array}$$

where *cov*(.) and *var*(.) denote the covariance and variance functions, respectively.

### Channel Pair Selection

The connectivity is a measure calculated between two channels, so the main question is how to find channel pairs that contain the most information related to the hand motion. To address this problem, we employed a brute-force approach to find electrode pairs that represent the highest correlation with movement signals. The brute-force approach is based on searching the whole space of features. In this case, there was $\left(\frac{56}{2}\right)=$ 1,540 different channel pairs. The training dataset for each subject was used for the brute-force search algorithm. PLV or MSC features based on each channel combination were calculated. There were 30 frequency bands and six time-lags, so a total of 30 × 6 = 180 features were extracted from each channel pair. Regression analysis for each pair was performed based on five-fold cross-validations (inner CV). The channel pairs were ranked based on the highest Pearson correlation values. The algorithm was implemented with different numbers of selected channel pairs ranging from 10 to 40. The best result was achieved with 20 channel pairs. Hence, for the rest of the study, we selected 20 channel pairs, each corresponding to the highest positive correlation values alongside each movement axis, resulting in 40 channel pairs per subject.

### Feature Selection

Selecting channel pairs highly correlated with movement shrinks the feature space. However, concatenating features from pairs chosen leads to a huge feature space. It contains 40 channel pairs where each of them represents 180 features. So the whole feature matrix includes 40*180 = 7,200 columns, which may represent redundant information for decoding hand movement. The goal of the feature selection algorithm is to select the most relevant features to hand movement. This study exploits a two-stage feature selection algorithm (Robinson et al., 2015). The features representing higher Pearson correlation with hand motion are selected at the first stage. These features can be from various channel pairs, frequency sub-bands, or time lags. Then, a regression-based predictor subset selection by a backward elimination process is employed (Myers, 1990). This approach is common in MLR models to obtain the best fitting combination of variables. The advantage of backward elimination is that it considers the joint predictive capabilities of predictors. The description of backward elimination is as follows: the MLR model is built with a set of features based on Equation (10). The regression weights vector is calculated *via* Equation (11). Then, the least significant feature (with highest *p*-value) is removed from the feature matrix. After that, the model is re-calculated with remaining set of features. This process is repeated until the optimum number of features is achieved. In summary, at the first stage, predictors showing high correlation with hand movement are selected, then the regression model will be fine-tuned from statistical point of view.

## Results

**Figure 4:**
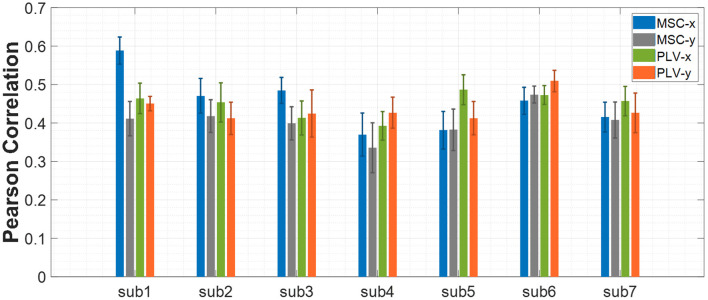
Decoding performance of center-out trajectory reconstruction for MSC and PLV features. The bar plots represent the mean ± SD of the Pearson correlation coefficients for each feature alongside each axis.

The procedure of feature extraction, channel pairs selection, and feature selection were discussed in the previous sections. In this section, the results obtained from the proposed approach are presented. As we stated earlier, the first step of processing data from each subject was to find the best channel pairs for each feature type (PLV or MSC) separately. Then, the best 40 channel pairs have been used for feature extraction. The next step was to apply the proposed feature selection method to find the most related features to hand movement. Experimental results revealed that 300 features would lead to optimum results for all subjects, so the number of features selected *via* the proposed algorithm was set to 300 throughout the results. The Pearson correlation coefficient has been calculated and averaged between predicted and actual movement based on ten repetitions of nested CV for all data blocks of each subject. Figure 4 shows the mean ± SD of Pearson correlation coefficients for all seven subjects. The results for the x-axis and y-axis are reported separately.

**Figure 5:**
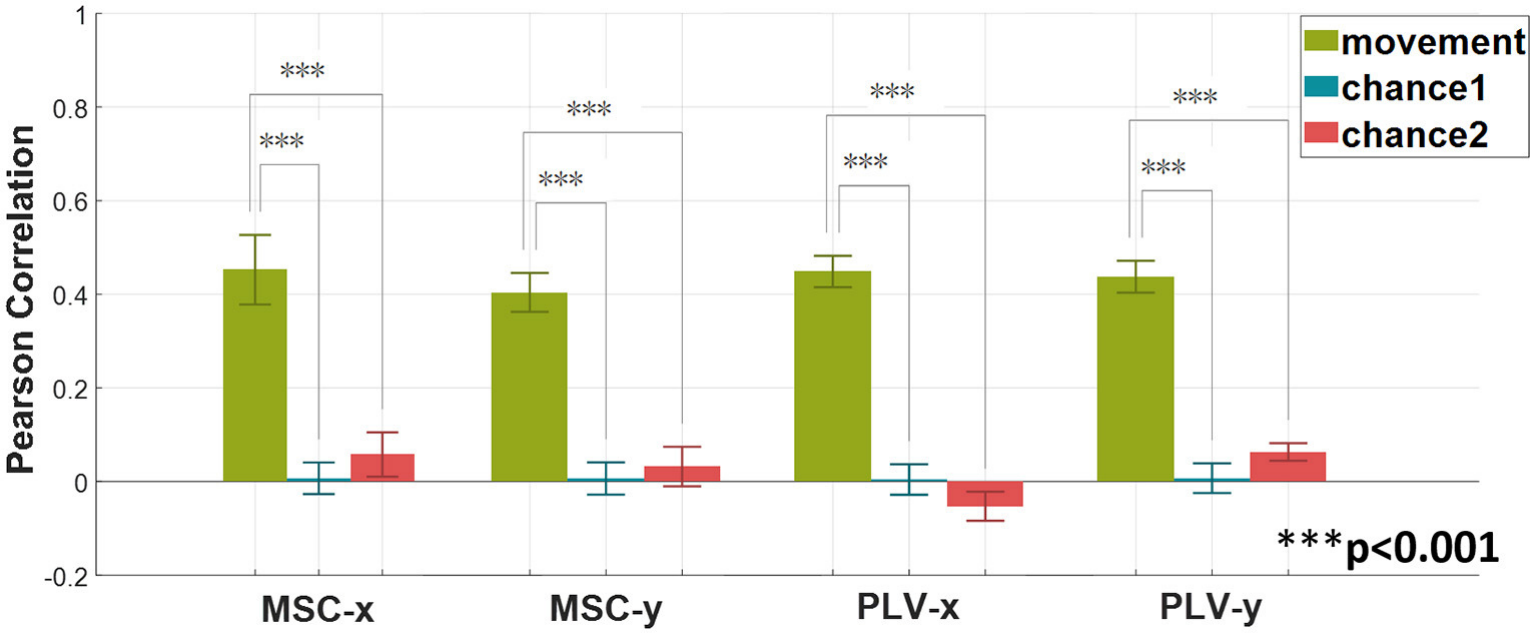
Verifying the statistical significance of the proposed method with MSC and PLV features as regression predictors *via* Wilcoxon rank-sum test. Two surrogate methods are used for calculating chance levels. The method denoted by chance1 is based on shuffling the target response. The second method indicated as chance2 is based on shuffling EEG signals. The above graph shows the average (mean ± SD) results over all subjects (Wilcoxon rank-sum test, ***p < 0.001).

In order to verify the statistical significance of results in Figure 4, two surrogate approaches for chance level calculation are employed. The first approach randomly shuffles the movement data (Vidaurre et al., 2019). This approach is denoted by chance1. The second approach randomly assign recorded EEG signals to movement profiles (Antelis et al., 2013; Úbeda et al., 2017). In this method, the movement data remains intact. Chance2 denotes this approach. The Wilcoxon rank-sum test is employed to verify the statistical significance of the proposed method against the chance level. The results–averaged on all subjects- are summarized in Figure 5.

The average of Pearson correlations for MSC features along the x-axis, and y-axis for all subjects equal 0.44 and 0.40, respectively. Similarly, the average Pearson correlation coefficients for PLV features along the *x*-axis and *y*-axis are equal to 0.44 and 0.43, respectively.

**Figure 6:**
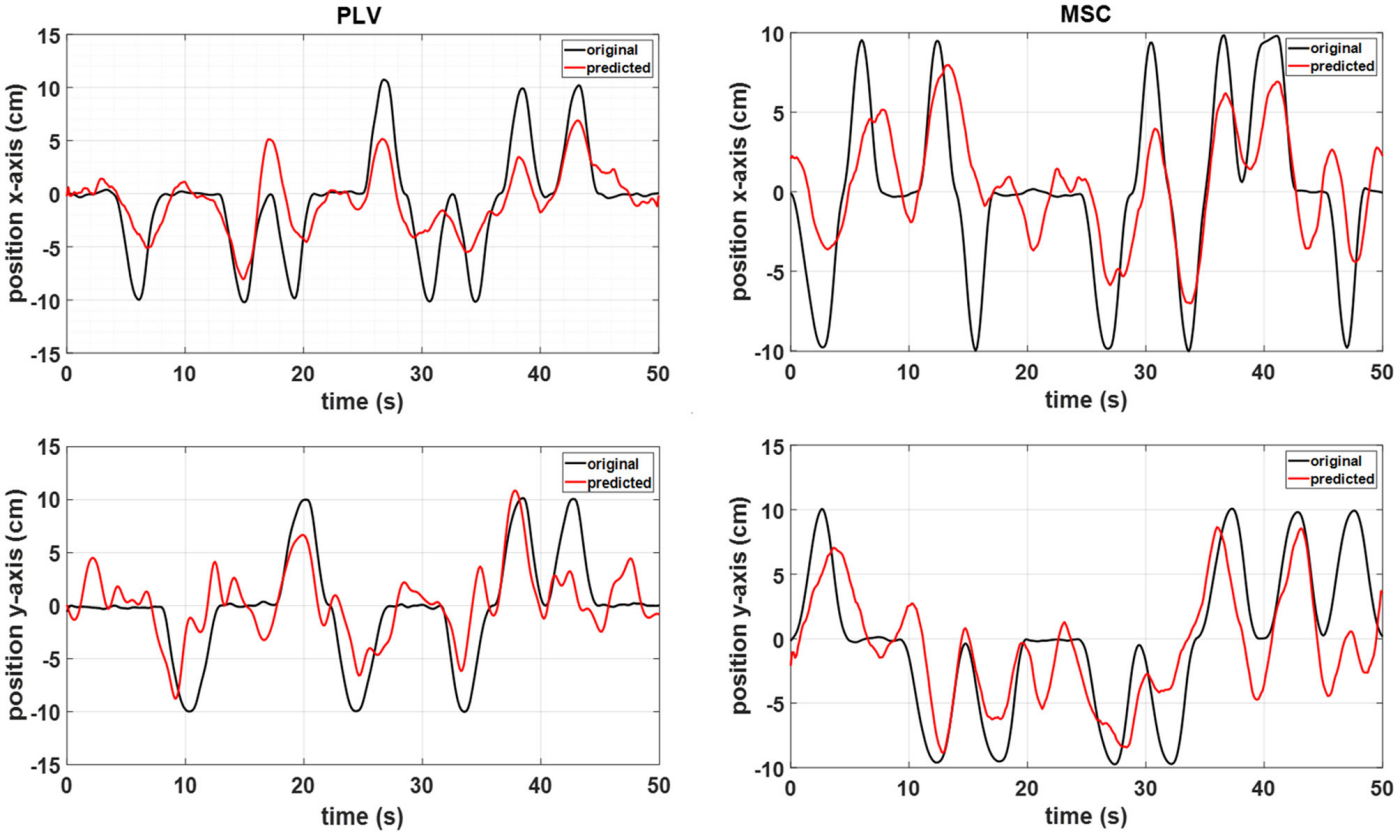
A representative sample of the MLR reconstructed movement positions with the PLV features (left) and MSC features (right) from subject 6 data.

Representative hand position reconstruction results with PLV and MSC features for a single subject are presented in Figure 6. The hand trajectory reconstruction method is able to follow the variations of actual movement.

**Figure 7:**
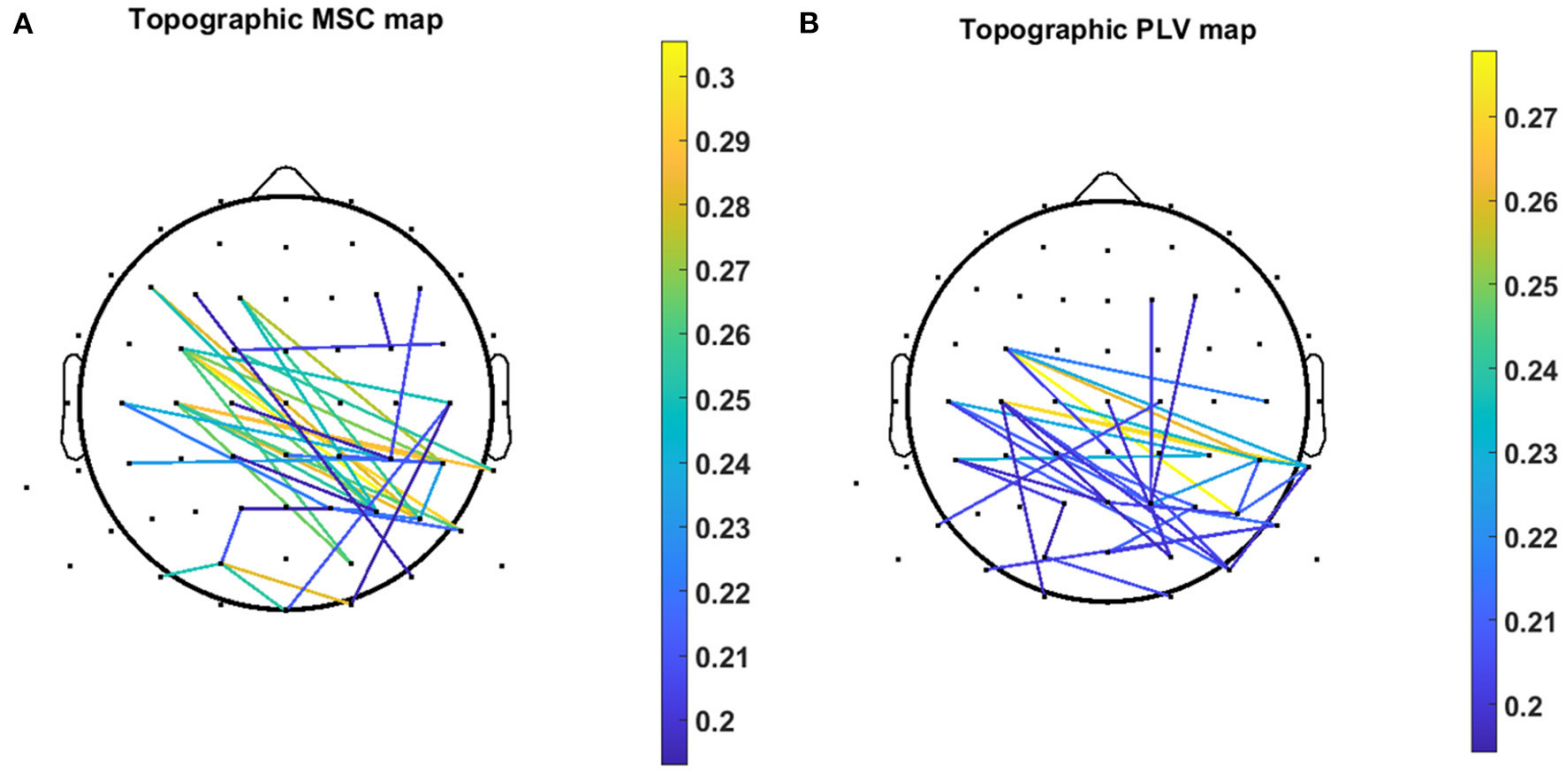
Topographic map of 40 channel pairs with highest PCC over all subjects for **(A)** MSC features and **(B)** PLV features. Color bar represents the value of the PCC related to the specific channel pair.

Figure 7 demonstrates the scalp locations of 40 pairs of channels which show the highest contribution in regression decoding for MSC and PLV features over all subjects. The color bar demonstrates the PCC of each single channel pair calculated in a five-fold inner CV.

In order to investigate the contribution of each frequency band in movement reconstruction, the regressor weights have been analyzed. The formula below describes the contribution of *i*^*th*^ frequency band in movement reconstruction based on the regression weight vector (Khorasani et al., 2016).

(13) $$\begin{array}{r} \%C_{freq}\left(i\right)=\frac{\sum_{c=1}^{N_{c}}\sum_{\tau =0}^{N_{\tau }-1}|\beta_{ci\tau }|}{\sum_{c=1}^{N_{c}}\sum_{i=1}^{N_{f}}\sum_{\tau =0}^{N_{\tau }-1}|\beta_{ci\tau }|} \end{array}$$

**Figure 8:**
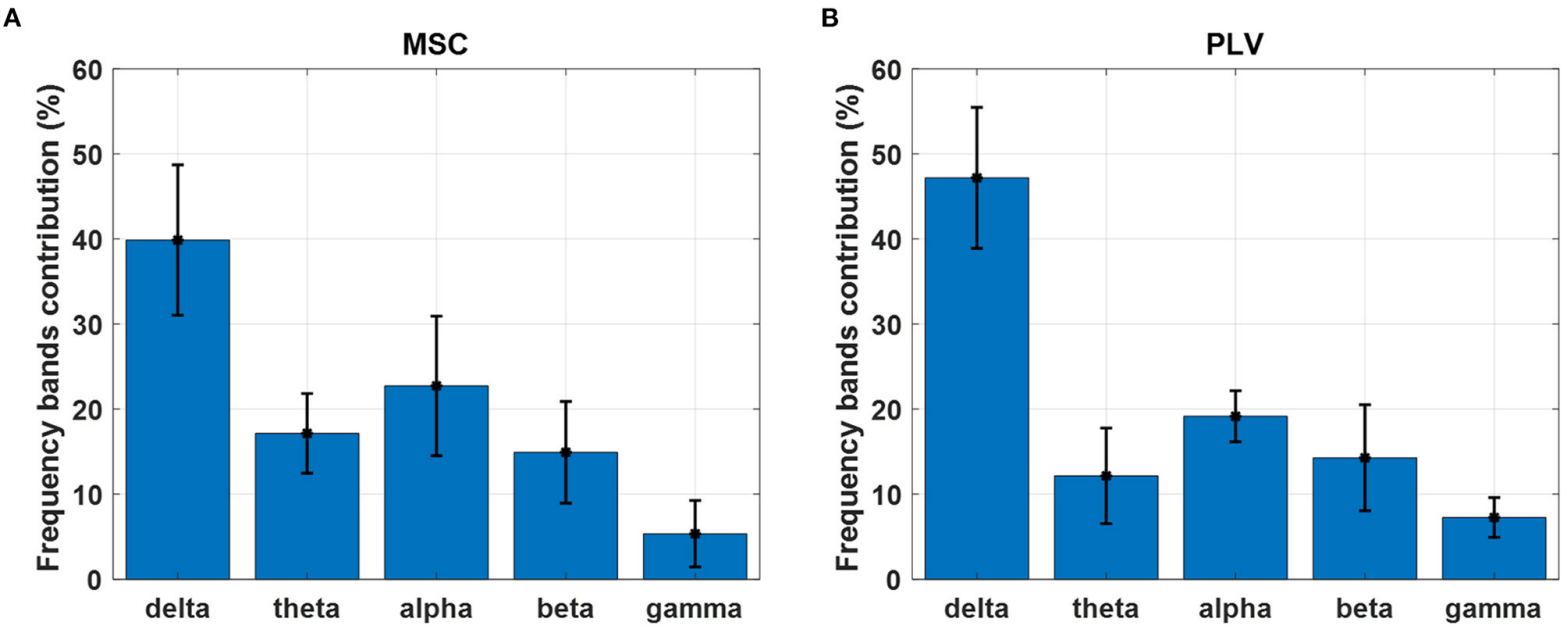
Contribution of each frequency band in movement decoding with **(A)** MSC and **(B)** PLV features. Each bar displays contribution weights' mean and standard deviation in each frequency band.

The percentage of frequency contributions has been calculated and averaged over delta (1–4 Hz), theta (4–8 Hz), alpha (8–14 Hz), beta (14–30 Hz), and gamma (30–45 Hz) frequency bands. The results are depicted in Figure 8 for MSC and PLV features. The delta and alpha bands show a higher contribution in motion decoding for PLV and MSC features. The features from gamma-band have the least contribution in movement decoding.

## Discussion

Estimating kinematic parameters of hand movement is one of the main goals in the BCI research field. Numerous studies have investigated this subject from both invasive and non-invasive modalities. In the realm of non-invasive BCIs, The first attempts focused on decoding hand movement directions as a discrete parameter (Hammon et al., 2007; Waldert et al., 2008). However, the continuous nature of hand motion requires continuous decoding of kinematic parameters. Therefore, the new generation of BCIs aims to decode continuous movement parameters such as position and velocity (Bradberry et al., 2010; Korik et al., 2018). The motion trajectory decoder BCIs are generally based on features extracted from the amplitude of brain signals. At the same time, a type of feature extraction that relies on bivariate connectivity analysis is usually ignored. The main objective of this study was to explore the feasibility of using phase-based connectivity features in predicting hand position from EEG signals. We proposed PLV and MSC-based feature extraction methods to reconstruct hand trajectory. First, some studies investigating the phase-based connectivity during hand movement tasks are summarized in this section. Then some interpretations and rationale of our study are presented. Next, our regression results are compared to two other methods. Finally, the strengths, limitations, and future works are summarized.

### Phase-Based Connectivity in Hand Movement Tasks

The phase-based connectivity features are widely used to analyze and interpret the functional relationship between brain regions. There are several studies that investigate functional brain connectivity during upper limb movement tasks. Ford et al. (1986) studied the EEG coherence during a fist-clenching and finger extension task of right/left or both hands. They found that MSC increases especially in the 9–12 Hz band during hand movement conditions compared to the rest state. The MSC changes were more apparent in the prefrontal, premotor, and motor areas. The event-related coherence was investigated by Leocani et al. (1997) in a self-paced right index finger movement task. They found that the magnitude and phase of coherence in 10 Hz and 18–22 Hz EEG signals in the frontal lobe are involved in movement planning and execution. Manganotti et al. (1998) studied the task-related MSC in a sequential finger movement that involved simple to complex movements. They found an increase in task-related MSC over frontocentral regions and a decrease in temporal and occipital areas. For all movement sequences, the increase of MSC in alpha and beta band compared to the rest state was the largest in the electrode pairs overlying both hemispheres' frontal, central and parietal regions. A decrease of MSC occurred mostly for electrode pairs overlying the temporal, occipital, and prefrontal regions. Santos Filho et al. (2009) investigated the MSC for detection of event-related potentials related to index finger movement execution and imagination task. They found that MSC in the delta band is related to the detection of movement execution or imagination. There are also some research studies investigating PLV in motor tasks. Popovych et al. (2016) found phase locking in delta–theta frequency band (2–7 Hz) in motor areas prior to and at the onset of movement execution. PLV features from EEG signals were also used in the binary classification of hand movement in a center-out task in work by Chouhan et al. (2018). They found that features at low delta play a significant role in the classification of hand direction. In addition, including features from theta and alpha bands increased the classification accuracy significantly.

In this work, we exploited MSC, and PLV features as predictors for linear regression to decode continuous hand movement. Figure 7A demonstrates the MSC topography of channel pairs with the most contribution in hand movement decoding. The channel pairs overlay centroparietal and frontoparietal areas show the most contribution in regression. These topographic results are in agreement with Leocani et al. (1997) and Manganotti et al. (1998) that report MSC changes in these areas during movement. Figure 7B shows the topographic map of PLV features. The most important channel pairs for PLV features overlay central and parietal areas. Figure 8A displays the contribution of each frequency band in regression using MSC features. The decoder algorithm has been used MSC features mostly in delta and alpha bands. This is generally in agreement with results in Ford et al. (1986), Manganotti et al. (1998), and Santos Filho et al. (2009). For PLV features, the decoder has mostly selected features in the delta band (Figure 8B) which corroborates the results in Chouhan et al. (2018).

The rationale behind using phase-based connectivity in this study can be explained as follows: from a mathematical perspective, phase and power are mostly independent signal measures (Cohen, 2014). Consequently, the phase-based and power-based connectivity can reveal different patterns of a neural phenomenon. Typically, phase and power reflect different neurophysiological dynamics. Phase mostly indicates the timing of the activity within a neural population, and power generally reflects the spatial extent of the neural population (Cohen, 2014). Therefore, employing the phase-based features can address the problem of continuous decoding from a new perspective. The advantage is that the phase-based features are related to the timing of neural activity. So, the features such as PLV would be an appropriate choice as predictors. The second connectivity measure used throughout our analysis is MSC. It can be seen as a PLV measure in which the power values weigh the phase values. Hence, the MSC measure relies on both the amplitude and phase of signals.

The MLR method was exploited to estimate the hand position in this study. The reason behind this selection is that in situations with a low signal-to-noise ratio (such as EEG recordings) or a small number of training cases, the MLR can outperform non-linear methods (Hastie et al., 2009). In addition, the parameters of linear models are neurophysiologically interpretable. It means that significant nonzero regression weights are only observed at channels which their activities are related to the brain process under study (Haufe et al., 2014). The disadvantage of MLR is that it is heavily susceptible to overfitting problems in the presence of a large number of features. Thus, a channel pairs selection and a feature selection algorithm have been employed to reduce the size of feature space and prevent the linear model from overfitting.

### Comparison of the Proposed Method to Other Approaches

In this part, we compare our results to two recently proposed approaches: first, a method proposed by Zeng et al. (2019) has been exploited to decode the hand movements of our center-out task. Zeng et al. have shown that using the phase of low-delta EEG as predictors would outperform the results of amplitude features in a center-out reaching task. The method is based on using the phase of low-delta 0.1–1Hz EEG signals and their time lags as predictors for linear regression; for more details, see Zeng et al. (2019). The results of applying the Zeng method to our data are summarized in Table 1.

**Table 1:** Comparing reconstruction performance of the proposed method to Zeng's and BTS methods.

	**Zeng's method** **(Zeng et al.**, **2019****)**		**BTS method** **(Korik et al.**, **2018****)**		**PLV**		**MSC**	
	***x*-axis**	***y*-axis**	***x*-axis**	***y*-axis**	***x*-axis**	***y*-axis**	***x*-axis**	***y*-axis**
sub1	0.32 ± 06	0.27 ± 07	0.39 ± 08	0.32 ± 07	0.46 ± 0.03	**0.45 ± 0.02**	**0.58 ± 0.03**	0.41 ± 0.04
sub2	0.22 ± 04	0.19 ± 03	0.31 ± 0.07	0.24 ± 0.06	0.45 ± 0.05	0.41 ± 0.04	**0.47 ± 0.04**	**0.42 ± 0.04**
sub3	0.11 ± 06	0.21 ± 0.5	0.26 ± 0.08	0.22 ± 0.06	0.41 ± 0.04	**0.42 ± 0.06**	**0.48 ± 0.03**	0.39 ± 0.04
sub4	0.26 ± 0.04	0.12 ± 0.04	0.31 ± 0.04	0.33 ± 0.05	**0.39 ± 0.03**	**0.42 ± 0.04**	0.36 ± 0.05	0.33 ± 0.06
sub5	0.12 ± 0.04	0.20 ± 0.04	0.38 ± 0.03	0.31 ± 0.03	**0.48 ± 0.03**	**0.41 ± 0.04**	0.38 ± 0.04	0.38 ± 0.05
sub6	0.20 ± 0.08	0.11 ± 0.06	0.26 ± 0.1	0.16 ± 0.07	**0.47 ± 0.03**	**0.50 ± 0.03**	0.45 ± 0.04	0.47 ± 0.02
sub7	0.19 ± 0.08	0.18 ± 0.06	0.28 ± 0.09	0.29 ± 0.07	**0.45 ± 0.04**	**0.42 ± 0.05**	0.41 ± 0.04	0.40 ± 0.05
average	0.20 ± 0.07	0.18 ± 0.05	0.31 ± 0.05	0.27 ± 0.06	**0.44 ± 0.03**	**0.43 ± 0.03**	0.44 ± 0.07	0.40 ± 0.04

*The best result on each axis is bolded for each dataset*.

Using the low-delta phase features as predictors in our center-out task led to inferior results compared to our proposed methods. We speculate it is due to the differences in tasks and analysis intervals. The movement period which was analyzed in Zeng's paper was in the 0–0.2 s interval relative to movement onset. It was equal to their shortest trial. The center-out task in Zeng's paper is just focused on moving from the home position to the target. In contrast, the length of trials in our study is not equal. Additionally, as we said earlier, the movement in our task comprises three phases. We believe that the Zeng method failed in our study because our task trials are much longer with different lengths and entail forward and backward movement.

The second approach used as an alternative method is band power time-series (BTS) proposed by Korik et al. (2018). The BTS method calculates power time series in six different sub-bands of EEG signal using a 500 ms time window. The task in the Korik study was a 3D reaching task where a subject sitting on a comfortable chair had to generate pointing movements with his right hand to four targets distributed in 3D space in synchrony with an auditory cue. We exploited the BTS method as described in Korik et al. (2018) to extract features from different frequency bands and then the 300 best features selected *via* our proposed feature selection technique. The results of the BTS method on our data are presented in Table 1. This method can achieve the average correlation coefficient of 0.29 on both axes. The results in Table 1 demonstrate that the proposed methods improve the average of Pearson correlation coefficient on both axes over all subjects.

**Figure 9:**
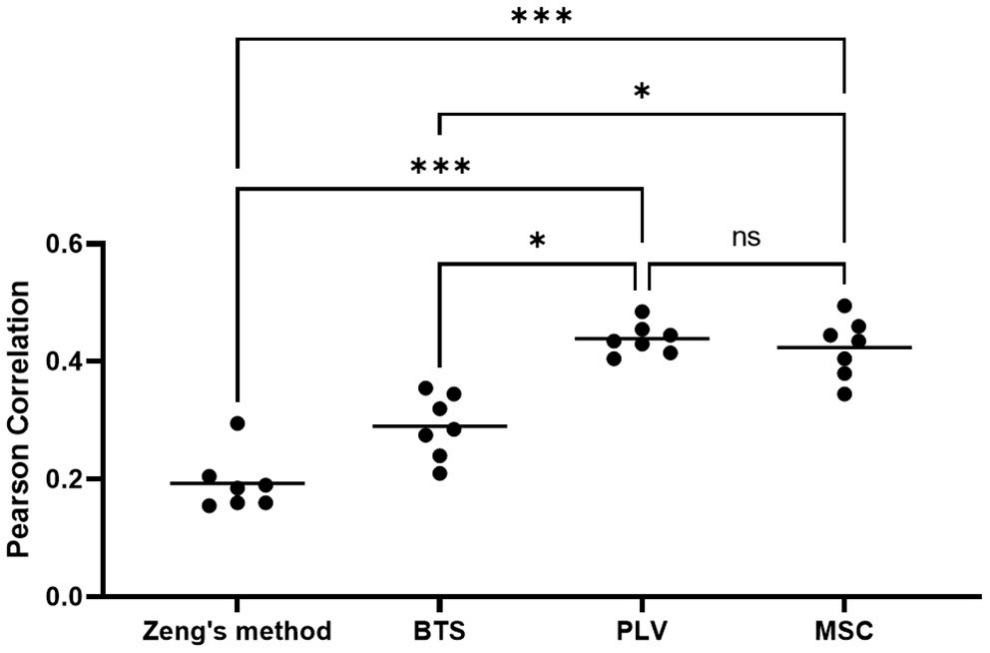
The result of Friedman ANOVA test with FDR correction. Each dot indicates the average of PCC for each subject. The horizontal bars display the mean of each group (*p < 0.05, ***p < 0.001, ns, not significant).

To evaluate the statistical significance of proposed methods, a one-way Friedman ANOVA test (*n* = 7, *k* = 4, *p* < 0.0003) followed by Benjamini et al. (2006) correction for multiple comparisons has been applied to the average Pearson correlation coefficient of each subject in four methods. The statistical test result is presented in Figure 9, which is produced using Prism GraphPad 9. In a nutshell, our result reveals that in a center-out movement task with variable length trials, the phase-based connectivity features that address neural activity timing can achieve statistically superior results.

### Strengths, Limitations, and Future Works

Here we briefly summarize the strengths of the study. First, using the optical motion tracker allows the subjects to perform the task naturally without using manipulandum or exoskeleton, which may introduce inconvenience to the movement. Second, the subjects did perform the task at their own pace without any limitation. In fact, the task was not synchronized with any auditory or visual cue to make the length of trials equal. To reduce the effect of muscle artifact on EEG recording, the participants were just advised to do the task at a desirable slow pace. The next strength of this work is the usage of phase-based connectivity features to decode continuous movement in an EEG center-out task. This approach has led to superior results compared to amplitude and phase-based methods.

The usage of connectivity features is not without caveats. Because the connectivity measure is naturally defined between two signals, the dimension of feature space for a multi-channel recording will become colossal. This issue has been addressed with a channel pairs selection technique. The brute-force algorithm exploited to find the best channel pairs is time-consuming because it considers all possible combinations of channel pairs. In fact, the computational complexity escalates as the number of recording channels increases. One possible way to improve the time efficiency of brute-force search is to reduce the size of search space by selecting a relevant subset of recording channels before channel-pair selection.

The second limitation is that the nature of the center-out task is restricted. In other words, the hand movement is limited to reaching four orthogonal targets in the horizontal plane. We are working on a future study based on a pursuit tracking task in which the hand is freely moving in a 2D horizontal plane.

To date, most studies on decoding continuous hand movement from EEG signals rely on extracting features from the amplitude of recording channels, and the connectivity features are mostly ignored. This work reveals that using PLV and MSC features from EEG recordings can be considered as a competent features set for decoding hand movement trajectories in a center-out task. The overall results of all subjects suggest that using phase-based connectivity features would improve the hand trajectory reconstruction on our data compared to two recent methods based on amplitude or phase feature extraction. This new approach could be exploited in designing new types of BCIs that use information between recording channels to increase the reliability and accuracy of predicting hand motion trajectories.

## Data Availability Statement

The raw data supporting the conclusions of this article will be made available by the authors, without undue reservation.

## Ethics Statement

The studies involving human participants were reviewed and approved by Ethics Committee of Iran University of Medical Sciences with approval ID IR.IUMS.REC.1400.382. The patients/participants provided their written informed consent to participate in this study.

## Author Contributions

SH and VS designed and conceptualized the project. SH performed the experiment, analyzed the data, and wrote the manuscript. VS reviewed and revised the manuscript, and supervised the research. All authors contributed to the article and approved the submitted version.

## Conflict of Interest

The authors declare that the research was conducted in the absence of any commercial or financial relationships that could be construed as a potential conflict of interest.

## Publisher's Note

All claims expressed in this article are solely those of the authors and do not necessarily represent those of their affiliated organizations, or those of the publisher, the editors and the reviewers. Any product that may be evaluated in this article, or claim that may be made by its manufacturer, is not guaranteed or endorsed by the publisher.
